# Resources and Relationships for Success: Perspectives from Students, Alumni, and Staff of a High School-Based Parenting Program

**DOI:** 10.1007/s10995-026-04260-5

**Published:** 2026-05-11

**Authors:** Libby Hladik, Clara Mack, Sophia Banez, Marie Hynek, Lan Vo, Jessie Loeb, Stephanie Gramann, Karla K. Ausderau

**Affiliations:** 1 Department of Kinesiology, Occupational Therapy Program, University of Wisconsin–Madison, Madison, WI 53706 USA; 2 Capital High School Parenting Program, Madison Metropolitan School District, Madison, USA; 3 Waisman Center, University of Wisconsin–Madison, Madison, USA

**Keywords:** Expectant and parenting teens, School-based support, Social support, High school parenting program

## Abstract

**Objectives:**

High school-based parenting programs have been identified as a way to support teen mothers as they manage their complex roles as parent and student. The purpose of this study was to describe components within a high school-based parenting program that support the overall success of the mother and child through the perspectives of current students, program alumni, and staff.

**Methods:**

We conducted a phenomenological study using semi-structured interviews with current students (*n* = 8) and program alumni (*n* = 11) from one high school-based parenting program and staff from seven high school-based parenting programs (*n* = 12). Thematic analysis was used to identify essential program supports within and between the three different groups. Member checking was completed to verify that the final themes represented the experiences shared in the interviews.

**Results:**

Four themes were identified supporting the success of teen mothers in high school-based parenting programs: Things That Make the Program Run, Basic Needs, Classroom Supports, and Connections Outward. Themes were found across all participants with comparison between groups revealing nuances in their experience and perception of supports.

**Conclusions for Practice:**

High school-based parenting programs need to move beyond providing traditional supports to focus on facilitating social connections, ensuring the basic needs are met, and utilizing non-traditional learning strategiesnull. Programs should name and measure strategies used to measure program effectiveness to understand the academic, health, and well-being implications for both parent and child short- and long-term.

## Significance

This study highlights the critical role of high school-based parenting programs in supporting pregnant and parenting teens, particularly by meeting basic needs, fostering social connections, and offering flexible, non-traditional academic support. It emphasizes the importance of addressing both immediate and long-term needs from the perspectives of students, alumni, and staff. By focusing on often-overlooked social and emotional supports alongside educational accommodations, this study extends existing literature and calls for programs to be evaluated not just on academic outcomes, but also on health, well-being, and intergenerational impact.

## Introduction

Teen mothers are a unique population who take on dual roles as both students and parents requiring support to fulfill these roles. Although teen birth rates have decreased by 73% since 1992, the U.S. teen birth rate remains the highest among developed countries (Martin et al., 2021). Additionally, Hispanic and non-Hispanic Black populations face teen birth rates that are more than double compared to their non-Hispanic White counterparts (Osterman et al., 2024). In the state of Wisconsin, the average teen birth rate has declined overall but continues to exhibit persistent racial disparities (Hoffner et al., 2024) and is higher than the national average in non-Hispanic Black, Hispanic, and Asian populations (CDC WONDER, 2020). Understanding supportive factors that positively influence the health and well-being of teen mothers, particularly those of color, from their own perspectives is essential for improving outcomes for both the mothers and their children.

Teen mothers face many individual and systemic challenges as parents and students. The dual role requires teen mothers to balance the high demands of schoolwork and peer relationships while learning how to parent (Harding et al., 2020b; Washington, 2020). Balancing complex demands can be difficult, leading to high school dropout or not pursuing secondary education (Berthelon et al., 2025; Pinzon et al., 2012; Schulkind & Sandler, 2019). Socioemotionally, teen mothers often experience high levels of depression and stress that have been shown to have a negative impact on child development (Huang et al., 2014; Laurenzi et al., 2020). Additionally, teen mothers of color face additional systemic barriers with limited access to contraception and reproductive healthcare (Galloway et al., 2017). An opportunity for intervention to reduce barriers for this vulnerable population is through high school-based parenting programs (Harding et al., 2020a). Parenting programs in high schools provide specific education and connections to social services for teen mothers to address known challenges for teen parents and support the transition through childbirth into parenting.

High school-based parenting programs are increasingly recognized as an intervention to improve the educational outcomes of teen parents and their children (Asheer et al., 2020; Margolis et al., 2020; Mytton et al., 2014). Providing teen mothers with individualized academic support, flexible scheduling, and courses focused on postsecondary education in a high school-based parenting program improves academic success (Asheer et al., 2020; Hernandez, 2020). In addition, parenting skill education, prenatal care, and early childhood development classes improve resilience and self-sufficiency in teen mothers (Asheer et al., 2020; Cox et al., 2019). Program evaluations for parenting programs in high schools have demonstrated that providing individualized support increases academic achievement, as well as improves parenting readiness, personal development, and mothers’ well-being (Asheer et al., 2020; Brouwer et al., 2019; Egan et al., 2020). Teen mothers report that social-emotional support, including supportive staff, support groups, and social activities, is important for engagement and success in parenting programs (Egan et al., 2020). To better understand program components that support student success, including both individualized academic and socio-emotional supports, firsthand experiences from program participants (i.e., students and staff) are essential.

Current literature suggests that high school-based parenting programs are working hard to implement individualized academic supports to facilitate positive outcomes for teen mothers and their children (Maslowsky et al., 2022; Morris, 2023). However, there are limited firsthand perspectives from students and staff as to what they identify as important within high school-based parenting programs to support their overall success beyond traditional educational services and outcomes. There also remains a lack of differentiation between how students and staff perceive the importance and impact of structured and unstructured program components. The purpose of this study was to describe components within a high school-based parenting program that support the overall success of the mother and child through the perspectives of current students, program alumni, and staff.

## Methods

This study was part of a community-based participatory program evaluation for an urban Wisconsin high school-based parenting program. A community-based participatory research approach was implemented where students, alumni, staff, and researchers collaborated on project development, goals, decision making, methods, and analysis (Collins et al., 2018; Tuck & Yang, 2014). The project protocol was determined exempt by the university ethics review board (2022-0315). The overall program evaluation aimed to develop recommendations based on the program’s primary goals to promote evidence-based practices for educating teen parents and supporting their children. The parenting program’s four main goals were to increase graduation rates, increase post-secondary enrollment, decrease repeat pregnancy, and increase parenting skills. This study was an early step in the program evaluation and used a phenomenological design with semi-structured interviews to gather the experiences and perspectives of students, alumni, and staff from local parenting programs.

### Participants

Participants (*n* = 31) included current students (*n* = 8), alumni (*n* = 11) who ranged from 2 to 20 years out of the program, local program staff (*n* = 6), and staff from surrounding programs (*n* = 6). Inclusion criteria for students and alumni involved current or past enrollment in the local high school-based parenting program. Recruitment focused on including individuals with a range of experiences within the program to capture a full view of possible outcomes. Staff members of the local program included current teachers, former teachers, a counselor, and special education assistants. Additionally, we recruited staff members from surrounding parenting programs (*n* = 6) to gain a broader view of what components support the success of teen parents. Inclusion criteria for all staff members were involvement with a high school-based parenting program. Before the interview, participants learned about the purpose of the program evaluation and interviews both verbally and in writing and were offered the opportunity to ask questions. Participation in the interview was voluntary.

### Procedures

#### Question Development

Semi-structured interview guides were developed collaboratively with students and staff who suggested initial topics and then reviewed questions. Open-ended questions focused on their participation in the program, program requirements, managing student and mothering responsibilities, challenges and facilitators to managing responsibilities, and perceptions of program resources.

#### Interview Procedures

Interviews were held in a private room at the program or via Zoom, lasting from 15 min to two hours, with an average of one hour. Participants provided verbal consent to have their interview audio recorded. Student and alumni interviewees were compensated with $20 cash for their participation. Interviews were transcribed verbatim, de-identified by the project team, and stored on a secure research drive using NVivo Software (2020) for data analysis.

### Data Analysis

Thematic analysis, as described by Braun and Clarke (2006) was used to analyze the interview transcripts. The coding team included six graduate students and one faculty member. In Phase 1, coders familiarized themselves with the data by listening to interview audio, before and during transcription. In Phase 2, coders independently identified prevalent ideas through open coding related to success as a student and mother. The team discussed common ideas to develop initial codes with definitions. The codebook was updated iteratively through independent coding, discussion, and code refinement. Using a finalized codebook, two graduate student coders independently coded each transcript. Codes were grouped into initial themes in Phase 3. A thematic map helped identify relationships between themes and identify primary and possible subthemes (Byrne, 2022). In Phase 4, researchers discussed how the themes were represented across participants and identified quotes supporting each theme. Initial themes were further examined for clarity and independence and collapsed as necessary. Finalized themes were named and defined in Phase 5.

Throughout data collection and analysis, measures were taken to ensure rigor and trustworthiness. Purposeful sampling was supported by program staff including recruiting participants who had expressed a range of positive and negative experiences in the program. Data saturation was achieved within the sample size as researchers began to hear similar ideas arise repeatedly (Saunders et al., 2018). Additionally, consensus coding was used to reduce potential bias between coders. The team employed reflexivity to acknowledge how prior experiences, reactions, and personal motives may influence their engagement with the data. To ensure accuracy and reduce bias, a member checking session was held with current students and staff to verify that the final themes represented the experiences shared in the interviews.

## Results

Students, alumni, and staff identified four themes supporting the success of teen mothers in high school-based parenting programs: (1) Things That Make the Program Run, (2) Basic Needs, (3) Classroom Supports, and (4) Connections Outward. Themes were found across all participants with some notable differences between the perspectives of students and alumni compared to staff, which is clarified within the theme descriptions and examples.

### Things That Make the Program Run

The first theme, Things that Make the Program Run, captured soft skills and human factors that were vital to supporting teen parents. Flexibility, promoting autonomy, fundamental recognition of parenting needs, guiding values such as anti-racist academic curriculum, responsive and individualized approaches, and space for adolescent identity formation were identified as necessary. Specifically, the anti-racist curriculum supported ongoing staff training that centered cultural humility, challenged systemic inequities in parenting and service systems, and promotes culturally responsive, strengths-based parenting practices. Students and alumni valued the program’s supportive infrastructure that enabled engagement in their responsibilities as students and parents. Our participants found it important to discuss the hard-to-define aspects of supporting teen parents. Many students and alumni shared stories of how staff acted respectfully and cared for students, recognizing their needs as students *and* as parents. Illustrating flexibility, an alum shared, “They like work with your schedule too, like if you’re sick or like you have a baby and the baby gets sick, and you got to have off. They’re like really understanding.” Students and alumni noted how less-tangible aspects of the parenting program bolstered their mental and social well-being while in the program and after graduation. Supportive relationships were built through a complex balancing of students’ personal needs and the requirements of the school and medical systems. Describing the approach and program values, one staff member shared, “Hopefully [all those resources] make parenting a little easier. Because there’s knowledge that’s shared, there’s education that’s shared, there’s love that’s shared, there’s support that’s shared. All that breaks down barriers.” Students and alums identified this theme in parallel to academic achievement, where staff considered it the glue that held the program together and an essential component of academic success. A staff member shared, “The other thing that makes a huge impact on the students is not… always like the academics, but it’s definitely the loving, supporting environment that’s created.” Students, alumni, and staff clearly valued the essential academic components of the program but also identified underlying social structures that were equally important for their success.

### Basic Needs

Basic Needs identified core necessities for students to focus on academic participation: transportation, affordable on-site childcare, mothering and infant supplies (e.g., nutritious snacks, diapers, wipes, formula), and safety. Reliable transportation and on-site childcare were imperative for school participation. A staff member noted, “When we had cabbing on a regular basis, we saw our kids so much more,” illustrating how transportation supported attendance. One alum recalled winter transportation challenges, “I remember one time I had to ride the bus to school with [my child] in my hands, and I’m just like, this is too much…” On-site childcare also reduced anxiety and supported academic focus. One student shared, “The fact that my daughter’s daycare is right down the hall is amazing because it was hard for me to bring her here. So, knowing that I can always check on her was amazing.” Another student noted, “Daycare helps a lot. Daycare helps so you get like a little time just to think about everything; you don’t have a baby climbing on you while you are trying to do your work.” Childcare and transportation facilitated consistent attendance but it was also essential for the teens to focus on their mother role during the school day while balancing academic demands.

Unique parenting resources meeting the basic needs of parent and child were essential to allow student parents to focus on school, which included a safe entrance to the high school and private space to feed their child during school. Safe spaces within the school environment supported both physical and psychological privacy and security, allowing parenting students to fully engage in their education. One staff member explained:

They need to feel safe coming to school, whether it’s for themselves and their own emotional well-being and knowing that no one’s going to judge them. And then they also need to feel safe bringing their children to schools or to, you know, their daycare or around.

A student described safety as, “I feel like it is a safe space as it is because it’s just women. I don’t know. I don’t know about other women, but I feel safer when it’s just women.” Another staff member emphasized: “We’re like in any other high school where there are fights and things that happen. And so that they have a separate entrance, you know, because they’re carrying baby… so we want to make sure that everybody is safe.” Although described in different ways, students and alumni valued the security the program provided through both the physical structures and emotional environment for them and their children.

While all participants noted the importance of Basic Needs, students and staff described them differently. Students and alumni focused on the immediate and direct impact of the resource on a day-to-day basis whereas staff described investing time and energy to ensure resources were available for the students throughout their time in the program. For example, staff worked to overcome transportation barriers that limited attendance by distributing gas cards and bus tokens, or by securing additional funding. One staff member shared, “I will try and write grants to get funding for like attendance incentives… if the student just is not coming on a bus and but somebody is driving them, we will give them a $25 gas card.” Staff described multiple ways that they relentlessly pursued or worked with administration to find the resources and advocate for the students to feel safe, have access to reliable transportation, and high-quality onsite childcare to allow them to focus their role as students.

### Classroom Supports

Classroom Supports encompasses academic services and specialized curriculum for parenting students, including individuated education support for general coursework and parenting and life skills training. Students valued personalized attention in smaller classes. One student explained:

Since it’s such a small group, like, you can get help way easier. They always, like, check in with each person to see, like, how you’re doing, which feels nice. Because I felt like when I was in big classrooms, I feel like I wasn’t really seen and that everybody understood but me.

Staff described their perspective on the developmental need for small groups and one-on-one attention for students. Describing why responsive and individualized teaching is necessary, a staff member noted:

Pregnant teenagers have no clue that they’re going to be parents… It’s your brain development at that age. You just, you cannot…think ahead. They don’t really understand that they’re going to be parents until they are parents. So having parenting education that is right there and then…is so helpful.

An alum described how specialized classes prepared her for motherhood: “It allowed me to mature more, and like, understand, well, it kind of like prepared me too for being a parent… I knew what to expect when I was going to give birth, so I wasn’t, like, more scared of that.” Discussing how classes extend to life skills beyond the immediate moment, a staff member shared:

We do financial literacy, and we talk about how to do your taxes. We talk about how to properly go in for a job interview. Even like what to wear and what to say and what to do and when should you be there? …we do a lot of the nutrition pieces… we do a lot about like with housing assistance, we do secondary education… We want them to be successful in the next stage of their life.

Students, alumni, and staff all described the importance of having the opportunity to learn in small classroom environments that provided additional time and responsive strategies to support their success. They equally valued the concurrent opportunity to engage in parenting and life skills coursework that supported their emerging needs in the program but extended after graduation.

### Outward Connections

The fourth theme, Outward Connections, captures critical relationships that began within the program but extended beyond graduation. Connections often began with meeting community partners during high school that would provide ongoing post-graduation support. One alumna valued connections to financial services, sharing:

Taking us to these guest speakers made me think about what I wanted to do. Like, if there was ever a time when I needed financial assistance or find, like I needed to find affordable housing, they were always giving me their information to contact them in the future and let me know that they will always be there as well.

Students and alumni described the long-term benefits of building connections that would extend beyond the program and staff intentionally fostered those opportunities for relationship-building with long-term benefits. An alum shared, “There was always good resources for us young mothers that didn’t know what to do after high school.” Allowing time and opportunity for social relationships to begin and be cultivated within the program created the foundation for lifelong support systems and success beyond high school.

## Discussion

Identifying necessary supports for pregnant and parenting teens within high school-based parenting programs is essential for the success of mother and child outcomes. The firsthand report of students, alumni, and staff in this study identified four primary aspects of high school-based parenting programs that contribute to successful outcomes: (1) Things That Make the Program Run, (2) Basic Needs, (3) Classroom Supports, and (4) Connections Outward. While traditional education components were mentioned, they were overshadowed by intangible social support and connections, meeting mother and infant basic needs, and providing additional learning strategies and education within traditional classrooms. While our findings do align with existing literature showing parenting programs provide necessary accommodation for pregnant and parenting students to complete high school (Asheer et al., 2020; Margolis et al., 2020; Mytton et al., 2014), this study extends and elevates the importance of providing a strong physical and psychological foundation in coordination with necessary accommodations and academic support to pregnant and parenting teens.

Things That Make the Program Run and Outward Connections emphasize vital but often overlooked and intangible aspects of high school-based parenting programs. Participants described how connections with peers, staff, and community partners created environments where they felt comfortable, cared for, and equipped for life beyond high school. Findings align with previous research that identified flexibility, responsive staff, and adapted curricula for parenting teens as essential for positive educational outcomes (Asheer et al., 2020; Brouwer et al., 2019). Egan and colleagues (2020) identified supportive staff and strong social connections as teen mothers’ most common reasons for continued involvement in high school-based parenting programs. Our study extends these findings, identifying flexibility, support of students’ autonomy, and safety as essential to developing supportive relationships responsive to students’ needs. Implementing a responsive approach and focusing on the essential but often intangible human aspects of a parenting program may improve parenting efficacy, graduation rates, and post-graduation outcomes and need to be measured in future research.

Basic Needs and Classroom Supports provide an understanding of foundational and concrete needs of pregnant and parenting high school students to attend school and engage in learning while balancing their dual roles as parent and student. When basic needs were met, teen mothers shared that they could focus on school while caring for themselves and their children. Our findings are consistent with previous literature in identifying transportation, childcare, physical supplies, and individualized educational assistance as supportive to teen mothers’ well-being and high school participation (Brouwer et al., 2019; Egan et al., 2020; Harding et al., 2020a; Maslowsky et al., 2022). The anti-racist curriculum described by staff in our study highlights their focus on addressing systemic inequities for students and emphasizing regular training for staff to ensure the ongoing needs of teen parents are being met. Considering the short and long-term impacts of high school parenting programs that influence two generations is an important step in addressing racial disparities in teen parents (Burkhardt et al., 2022; Navarro-Cruz et al., 2021).

While similar themes were identified within students, alumni, and staff, nuanced but important differences were highlighted. For example, resources and relationships were recurring topics in all groups. However, students and alumni emphasized how these supports affected their current experiences within the program and staff focused on problem-solving to ensure their sustainability with adequate financial and staff resources. Staff viewed program impacts longitudinally invoking their experience and relationships with program alumni. They emphasized program components supporting long-term skill development, such as parenting and life skills. While alumni reflected their appreciation for the long-term impact, alumni and students reported it was the immediate day-to-day support of their needs that was important. Focusing on the immediate outcomes or benefits of the program is developmentally appropriate for teenagers experiencing a dramatic life transition to becoming a parent (Jamison & Feistman, 2021; Pinzon et al., 2012). Understanding these perspective differences allows programs to implement supports addressing both immediate and long-term needs.

The high school-based parenting program that was the focus of the evaluation used study findings as a foundation for structured reflection and program planning. They engaged in staff discussions to build on identified strengths, particularly the importance of supportive relationships, flexibility, and individualized approaches. They also consider important gaps in services such as transportation and community partnerships that have become more challenging due to funding constraints. Findings were leveraged and extended to inform successful grant applications, including efforts to expand programming into additional high schools and strengthen district-level supports for young parents. Although the study served primarily as foundational information for these initiatives, its impact was strengthened through continued collaboration and involvement of the research team within the parenting program and on related advisory boards.

The findings of this study should be considered in tandem with their limitations. Recruitment was primarily from one high school-based parenting program, potentially limiting applicability to other programs and regions due to the influence of local policies, rates of teen pregnancy, and funding mechanisms for parenting programs. Also, participants who opted to participate, particularly those with long and invested relationships with the programs, may skew towards positive associations and may not fully reflect all experiences. Future research should aim to recruit increasing diverse samples across parenting programs and begin to explore the crucial relationship between essential support identified in this study and long-term wellness, as well as traditional educational outcomes (e.g., graduation rates and post-secondary education) in pregnant and parenting teens.

## Conclusion

High school-based parenting programs need to move beyond providing traditional supports to focus on facilitating social connections, ensuring the basic needs of the mother and infant are met, and utilizing non-traditional learning strategies to foster long-term outcomes for both teen parent and child. While many programs may already be integrating findings from this study, they need to be named and measured to elevate and evaluate their importance in supporting traditional outcomes such as graduation outcomes and post-secondary enrollment. During program implementation and re-evaluation, the complexity behind frontline staff decision-making and execution must be considered, such as policies influencing funding, enrollment, and program delivery. Furthermore, future research should examine the effectiveness of high school-based parenting programs beyond academic outcomes and include health and wellbeing outcomes for teen mothers and their children.

## Data Availability

In order to protect the anonymity of participants, interview data will not be made publicly available.

## References

[CR1] Asheer, S., Zief, S., & Neild, R. (2020). Roadmap for effective school-based practices to support expectant and parenting youth: Lessons from the New Heights Program in Washington, DC. *Maternal and Child Health Journal*, *24*(S2), 125–131. 10.1007/s10995-020-02986-4 32737680 10.1007/s10995-020-02986-4 PMC7497361

[CR2] Berthelon, M., Contreras, D., Kruger, D., & Palma, M. I. (2025). Early maternity and paternity. Effects on educational trajectories. *Journal of Development Economics,**173*, Article 103404. 10.1016/j.jdeveco.2024.103404

[CR3] Braun, V., & Clarke, V. (2006). Using thematic analysis in psychology. *Qualitative Research in Psychology*, *3*(2), 77–101. 10.1191/1478088706qp063oa

[CR4] Brouwer, A. M., Foster, R. H., & Jalensky, A. (2019). An alternative school model for pregnant and parenting teens: A qualitative analysis. *Child and Adolescent Social Work Journal,**36*(5), 471–484. 10.1007/s10560-018-0575-z

[CR5] Burkhardt, T., Dasgupta, D., Schlecht, C., Carreon, E., & Pacheco-Applegate, A. (2022). Chicago Young Parents Program evaluation: Implementation evaluation and follow-up study. *Chapin Hall at the University of Chicago*. 10.1016/s0149-7189(02)00053-8

[CR6] Byrne, D. (2022). A worked example of Braun and Clarke’s approach to reflexive thematic analysis. *Quality & Quantity*, *56*(3), 1391–1412. 10.1007/s11135-021-01182-y

[CR7] CDC WONDER, Natality Public Use Files. (2020). *Teen births in Wisconsin*. America’s Health Rankings. https://www.americashealthrankings.org/explore/health-of-women-and-children/measure/TeenBirth_MCH/state/WI

[CR8] Collins, S. E., Clifasefi, S. L., Stanton, J., The Leap Advisory Board, Straits, K. J. E., Gil-Kashiwabara, E., Rodriguez Espinosa, P., Nicasio, A. V., Andrasik, M. P., Hawes, S. M., Miller, K. A., Nelson, L. A., Orfaly, V. E., Duran, B. M., & Wallerstein, N. (2018). Community-based participatory research (CBPR): Towards equitable involvement of community in psychology research. *American Psychologist,**73*(7), 884–898. 10.1037/amp0000167 29355352 10.1037/amp0000167 PMC6054913

[CR9] Cox, J. E., Harris, S. K., Conroy, K., Engelhart, T., Vyavaharkar, A., Federico, A., & Woods, E. R. (2019). A parenting and life skills intervention for teen mothers: A randomized controlled trial. *Pediatrics,**143*(3), Article e20182303. 10.1542/peds.2018-2303 30755464 10.1542/peds.2018-2303

[CR10] Egan, J., Bhuiya, N., Gil-Sanchez, L., Campbell, S., & Clark, J. (2020). Engaging expectant and parenting adolescents: Lessons from the Massachusetts pregnant and parenting teen initiative. *Maternal and Child Health Journal*, *24*(S2), 191–199. 10.1007/s10995-020-02880-z 31981063 10.1007/s10995-020-02880-z PMC7497382

[CR11] Galloway, C. T., Duffy, J. L., Dixon, R. P., & Fuller, T. R. (2017). Exploring African-American and Latino teens’ perceptions of contraception and access to reproductive health care services. *Journal of Adolescent Health,**60*(3), S57–S62. 10.1016/j.jadohealth.2016.12.006 10.1016/j.jadohealth.2016.12.006 PMC5571442 28235437

[CR12] Harding, J. F., Knab, J., Zief, S., Kelly, K., & McCallum, D. (2020a). A systematic review of programs to promote aspects of teen parents’ self-sufficiency: Supporting educational outcomes and healthy birth spacing. *Maternal and Child Health Journal,**24*(2), 84–104. 10.1007/s10995-019-02854-w 31965469 10.1007/s10995-019-02854-w PMC7497377

[CR13] Harding, J. F., Zief, S., Farb, A., & Margolis, A. (2020b). Supporting expectant and parenting teens: New evidence to inform future programming and research. *Maternal and Child Health Journal,**24*(S2), 67–75. 10.1007/s10995-020-02996-2 32860585 10.1007/s10995-020-02996-2 PMC7497376

[CR14] Hernandez, R. N. (2020). Providing the knowledge and empowerment for post-secondary advancement among teen mothers. *Journal of Student Affairs, New York University,**16*, 63–73.

[CR15] Hoffner, J., Vasquez, A., & Remington, P. (2024). Trends in teenage birth rates in Wisconsin, 2011–2022: Continued declines and persistent disparities. *WMJ: Official Publication of the State Medical Society of Wisconsin*, *123*(6), 460–463. 39908494

[CR16] Huang, C. Y., Costeines, J., Kaufman, J. S., & Ayala, C. (2014). Parenting stress, social support, and depression for ethnic minority adolescent mothers: Impact on child development. *Journal of Child and Family Studies,**23*(2), 255–262. 10.1007/s10826-013-9807-1 24653641 10.1007/s10826-013-9807-1 PMC3956110

[CR17] Jamison, T. B., & Feistman, R. E. (2021). Understanding teen parents in a modern context: Prenatal hopes and postnatal realities. *Prenatal Family Dynamics: Couple and Coparenting Relationships During and Post Pregnancy*. 10.1007/978-3-030-51988-9_16

[CR18] Laurenzi, C. A., Gordon, S., Abrahams, N., Du Toit, S., Bradshaw, M., Brand, A., Melendez-Torres, G. J., Tomlinson, M., Ross, D. A., Servili, C., Carvajal-Aguirre, L., Lai, J., Dua, T., Fleischmann, A., & Skeen, S. (2020). Psychosocial interventions targeting mental health in pregnant adolescents and adolescent parents: A systematic review. *Reproductive Health,**17*(1), Article 65. 10.1186/s12978-020-00913-y 32410710 10.1186/s12978-020-00913-y PMC7227359

[CR19] Margolis, A., Rice, T., Banikya-Leaseburg, M., Person, A. E., Clary, E., Zief, S., Adamek, K., & Harding, J. F. (2020). Meeting the multifaceted needs of expectant and parenting young families through the pregnancy assistance fund. *Maternal and Child Health Journal,**24*(S2), 76–83. 10.1007/s10995-020-02922-6 32385692 10.1007/s10995-020-02922-6 PMC7497367

[CR20] Martin, J. A., Hamilton, B. E., Osterman, M. J. K., & Driscoll, A. K. (2021). Births: Final data for 2019. National Vital Statistics Reports Volume. *National Center for Health Statistics*, *70*(2). 33814033

[CR21] Maslowsky, J., Stritzel, H., & Gershoff, E. T. (2022). Post-pregnancy factors predicting teen mothers’ educational attainment by age 30 in two national cohorts. *Youth & Society,**54*(8), 1377–1401. 10.1177/0044118x211026941 38107471 10.1177/0044118x211026941 PMC10723653

[CR22] Morris, S. R. (2023). Teen mothers forgotten: The gap between high school and higher education. *Journal of Higher Education Policy and Leadership Studies,**4*(2), 107–117. 10.61186/johepal.4.2.107

[CR23] Mytton, J., Ingram, J., Manns, S., & Thomas, J. (2014). Facilitators and barriers to engagement in parenting programs: A qualitative systematic review. *Health Education & Behavior*, *41*(2), 127–137. 10.1177/1090198113485755 23640123 10.1177/1090198113485755

[CR24] Navarro-Cruz, G. E., Dávila, B. A., & Kouyoumdjian, C. (2021). From teen parent to student parent: Latina mothers’ persistence in higher education. *Journal of Hispanic Higher Education,**20*(4), 466–480. 10.1177/1538192720980308

[CR25] NVivo. (2020). [Computer software]. QSR International Pty Ltd. https://community.lumivero.com/s/nvivo-knowledge-base?language=en_US

[CR26] Osterman, M. J. K., Hamilton, B. E., Martin, J. A., Driscoll, A. K., & Valenzuela, C. P. (2024). Births: Final data for 2022. National Vital Statistics Reports. *National Center for Health Statistics*, *73*(2). 10.15620/cdc:145588 38625869

[CR27] Pinzon, J. L., Jones, V. F., Committee on Adolescence, Committee on Early Childhood, Blythe, M. J., Adelman, W. P., & Schulte, E. E. (2012). Care of adolescent parents and their children. *Pediatrics,**130*(6), e1743–e1756. 10.1542/peds.2012-2879 23184113 10.1542/peds.2012-2879

[CR28] Saunders, B., Sim, J., Kingstone, T., Baker, S., Waterfield, J., Bartlam, B., Burroughs, H., & Jinks, C. (2018). Saturation in qualitative research: Exploring its conceptualization and operationalization. *Quality & Quantity*, *52*(4), 1893–1907. 10.1007/s11135-017-0574-8 29937585 10.1007/s11135-017-0574-8 PMC5993836

[CR29] Schulkind, L., & Sandler, D. H. (2019). The timing of teenage births: Estimating the effect on high school graduation and later-life outcomes. *Demography*, *56*(1), 345–365. 10.1007/s13524-018-0748-6 30607778 10.1007/s13524-018-0748-6

[CR30] Tuck, E., & Yang, K. (2014). R-words: Refusing research. In D. Paris & M. T. Winn (Eds.), *Humanizing research: Decolonizing qualitative inquiry with youth and communities* (pp. 223–248). Sage.

[CR31] Washington, S. E. (2020). Fostering empowerment through occupation: An overview of an urban school-based parenting program. *Journal of Occupational Science,**27*(2), 182–192. 10.1080/14427591.2020.1731844

